# The Role of Capsule Endoscopy in Crohn's Disease: A Review

**DOI:** 10.7759/cureus.27242

**Published:** 2022-07-25

**Authors:** Oladipo Odeyinka, Rasha Alhashimi, Sankeerth Thoota, Tejaswini Ashok, Vishnu Palyam, Ahmad T Azam, Ibrahim Sange

**Affiliations:** 1 Internal Medicine, University of Ibadan College of Medicine, Ibadan, NGA; 2 Internal Medicine, University of Baghdad College of Medicine, Baghdad, IRQ; 3 Internal Medicine, Meenakshi Medical College Hospital and Research Institute, Kanchipuram, IND; 4 Internal Medicine, Jagadguru Sri Shivarathreeshwara (JSS) Medical College, Mysore, IND; 5 General Medicine, Jagadguru Jayadeva Murugarajendra (JJM) Medical College, Davanagere, IND; 6 Internal Medicine, Allama Iqbal Medical College, Lahore, PAK; 7 Research, Karamshi Jethabhai (K J) Somaiya Medical College, Hospital and Research Center, Mumbai, IND

**Keywords:** pediatric crohn's disease, inflammatory bowel disease, crohn's disease (cd), colon capsule endoscopy, small bowel capsule endoscopy

## Abstract

Crohn’s disease (CD) is a chronic inflammatory disorder with a predilection for the small bowel. Although awareness of this disorder has increased over the years, it remains a diagnostic challenge for many physicians. This is exacerbated by the rising incidence and high recurrence rate following therapy in certain individuals. It is currently agreed that a multimodality approach is the best one, but with the advent of new modalities, that could be changing. Furthermore, given its impact on the mental health of patients and the cost of treatment, it is pertinent that we arrive at not only convenient but accurate modalities in its diagnosis and management. Among these investigative modalities is the relatively novel capsule endoscopy (CE) that not only provides a more patient-friendly alternative but avoids the need for invasiveness. Asides from its diagnostic capability, its influence on therapy and monitoring of known CD patients following treatment has been shown. This article has reviewed the current literature comparing the relevance of CE with other available modalities in diagnosing CD patients. We explored its therapeutic impact and how it influences monitoring post-treatment in CD. This article also discusses the complications of CE and the possible solutions to these complications in the future.

## Introduction and background

Crohn’s disease (CD) is a chronic inflammatory bowel disease (IBD) affecting the gastrointestinal tract, from the oral mucosa to the anus, with a particular predilection for the small intestine. It has been linked to low quality of life and a high rate of morbidity, and it frequently leads to problems that necessitate hospitalizations and surgical operations [1]. In the United States, about one to two million people suffer from IBDs, with a frequency of 70-150 cases per 100,000 people [2,3]. Presently, it is estimated that the incidence of IBDs has been increasing since the start of this century; this is particularly pronounced in newly industrialized countries [4]. An example of this is seen in China, where it went from a rare occurrence to a common one, accounting for a significant proportion of hospitalizations recently [5]. This rapid increase has been attributed to the fact that while it is incurable, it is hardly fatal [6]. The significant rise in the incidence and frequency of CD is also a cause for worry.

Prior to the sixties, the incidence of ulcerative colitis was much higher than that of CD, with the narrative changing over the recent years [2,3,7,8]. The incidence of CD is rising at a greater rate than other types of IBDs in Asia [9-11]. The frequency of CD peaks during the second and fourth decades of life, with a slight female predominance [12]. Based on the available data, the CD is considered a complex illness with a wide range of etiologies in which genetics and environment interact to cause disease [12]. While not much progress has been made regarding identifying the exact genetic variation that influences illness risk, about 200 genes have been linked to this condition [13]. Smoking and reduced fiber intake have been linked to the development of CD, smoking in particular with an earlier onset and worse disease prognosis/progression [14,15].

CD has a multitude of phenotypes or presentations: This is usually determined by the type, location, and disease severity [16]. This can pose a diagnostic challenge to physicians, given its varied symptoms (intestinal and extra-intestinal) with no particular pathognomonic feature. These symptoms vary from cardinal symptoms such as abdominal pain and diarrhea to extra-intestinal symptoms affecting diverse systems in the body. Therefore, to make a diagnosis, a combination of medical history, physical examination, and complementary diagnostic tests are employed [16]. Among these diagnostic tests is capsule endoscopy (CE), which provides a safe, patient-friendly, and sensitive method for determining the accurate diagnosis and monitoring disease activity [17]. Of worthy mention is the PillCam® SB3 capsule, the latest innovation in CE, with reduced reading time, improved diagnostic accuracy, and enhanced image quality [18,19]. It is crucial in CD, given its proclivity for the small intestine and the difficulty in assessing its length and tortuosity. Much has changed since it was first approved in 2001; with technological advancements, we are now able to do much more with CE [20,21]. This review article aims to explore a distinct perspective on the relevance of CE as a diagnostic tool in the management of CD and also to review the current developments and potential future opportunities in CE.

## Review

Role of capsule endoscopy in the diagnosis of Crohn’s disease

Clinicians have used endoscopic, histologic assessment and small bowel imaging to determine the extent of CD involvement. Cross-sectional imaging is now widely used in diagnosing CD as a result of advancements in imaging technology [22,23]. Nevertheless, it leaves the question to be answered - if CE alone is sufficient to aid in the diagnosis of CD. This is against the current guidelines that generally assume a gold standard of histopathology from ileocolonoscopy (IC) and/or surgery for diagnosis [16].

Some studies have compared CE with other radiological tests to determine specific parameters such as sensitivity, specificity, and accuracy, among others. These parameters are important biostatistical values that can help show a test's concordance with regard to a given reference, in this case - the gold standard of histopathology.

Bruining et al., in a multicenter, prospective study, compared CE with magnetic resonance enterography (MRE) and/or IC in a sample population of 99 patients with established CD [24]. They found that CE showed a high overall sensitivity in detecting active enteric inflammation in 94% of patients compared to 100% for MRE and/or IC. This was further improved in terms of specificity as CE was shown to detect accurately in 74% of patients enrolled compared to 22% with MRE and/or IC [24]. The above study's findings can be compared along the lines of a prospective study by González-Suárez et al. in 2018 [25]. Forty-seven patients with established or suspected CD were assessed, with 36 (76.6%) found to have small bowel lesions and less than half (21, i.e., 44.7%) detected by MRE (Table 1) [25]. Prichard et al. sought to investigate this prospectively in 38 established CD patients, 20 of whom completed the study [26]. The authors had widely the same results as the studies discussed earlier, with CE being more sensitive than MRE (80% vs. 60%, respectively) (Table 1) [26]. CE was also shown to detect more proximal and extensive small bowel disease. However, Wiarda et al., in their prospective study, had a contradictory report to the studies above [27]. Thirty-eight patients were included, with 13 being excluded due to suspicion of stenosis. It was reported that the specificity, sensitivity, and positive predictive value (PPD) of CE and MRE were 89% and 90%, 57% and 73%, and 67% and 88%, respectively (Table 1) [27].

**Table 1:** Summary of studies evaluating the diagnostic yield of capsule endoscopy CE: Capsule endoscopy; MRE: Magnetic resonance enterography.

References	Compared modality	n	Diagnostic yield of CE	Incremental yield of CE
Bruining et al. [24]	MRE	158	94	-6%
Prichard et al. [26]	MRE	38	80%	20%
González-Suárez et al. [25]	MRE	47	76.6%	31.9%
Wiarda et al. [27]	MRE	38	57%	-16%

These studies indicate that CE is indeed a reliable tool for assessment; however, it is often limited by stenotic lesions that prevent its passage and thus necessitate further invasive diagnostic modalities. The sample size of these studies, however, makes them statistically insignificant.

Pediatric population is a peculiar group that poses an area of interest, and this is because of specific physiological differences that distinguish them from the adult population. It stands to reason that since it does not involve general anesthesia, ionizing radiation, or deep sedation, it would be more readily accepted. In 2004, the FDA authorized CE as a diagnostic tool for children aged 10-18 years old, and in 2009, this age range was broadened to include children as young as two years old [28]. They have now become more extensively utilized by clinicians in the diagnosis of CD. A prospective study by Oliva et al. in 2016 compared CE with other modalities in a pediatric population of 40 patients [29]. It was found that the sensitivity, specificity, and CE values of small intestine contrast ultrasonography (SICUS) and MRE were lower than that of CE (90% and 83%, 85% and 89%,and 89% and 100%, respectively) [29]. Similarly, in a meta-analysis of childhood-onset IBD, CE had a diagnostic yield of 58%-72%, while ileocolonoscopy had a diagnostic yield of 0%-61% [30]. Several studies across the globe have assessed the cost-effectiveness of CE as a diagnostic tool. Levesque et al., in their study using a decision-analytic model, assessed the cost-effectiveness of CE as a diagnostic tool in CD [31]. It was concluded that it was not cost-effective after prior negative investigations such as ileocolonoscopy with a staggering quality-adjusted life-year (QALY) of $500,000 [31]. The above study can be contrasted with a study done by Saunders et al., which reported that CE is cost-effective and cost-saving with reduced costs, increased quality of life, and life expectancy [32].

Following the latest clinical guidelines, patients with a strong suspicion of CD and a negative ileocolonoscopy should undergo small bowel imaging [33]. The next step should be a CT enterography (CTE), MRE, or patency capsule when a stricture or obstruction is suspected. However, if there is a certainty of an absence of such, CE is recommended due to its relatively higher sensitivity.

Role of capsule endoscopy in the postoperative recurrence of Crohn's disease

Unfortunately, despite developments in the management and treatment of CD over the years, about 80% of patients still require surgery at some point, with approximately 70% developing postoperative recurrence within 12 months [34]. This is even further complicated as patients with these recurrences carry a worse prognosis [35].

To curtail these clinical relapses, it has been proposed that the bowel, particularly the neoterminal ileum, is examined six months after surgery [36,37]. Currently, ileocolonoscopy is said to be the gold standard in diagnosis and, thereby, monitoring of recurrence [16,38]. However, its invasive nature and need for sedation are unappealing, particularly to patients. Also, it is limited by its inability to visualize a part of the small intestine's distal portion.

CE is an alternative that has been used and tested in various studies. CE should ideally increase the visualization of the neoterminal ileum as the distorted anatomy post-surgical intervention can sometimes make intubation and viewing of the new terminal ileum problematic with ileocolonoscopy.

Beltrán et al., in a prospective study of 19 patients, found that while CE detected recurrence in 68% of patients, ileocolonoscopy diagnosed recurrence in about 25% of these patients (Table 2) [36]. It was also noted that CE picked up more small bowel lesions [36]. The distorted anatomy post-surgery explained this wide gap in the detection of recurrence. A similar prospective study that reported a different conclusion had been published by Bourreille et al. [39]. In 32 patients, they compared ileocolonoscopy (gold standard) with CE in the postoperative setting at a median of six months. In one patient, the neoterminal ileum was not visible during ileocolonoscopy, so he was disqualified. Ileocolonoscopy detected a recurrence in all but two of the 21 patients, with a sensitivity of 90% and a specificity of 100% [39]. In contrast, the sensitivity and specificity of the CEs were 62%-76% and 90%-100%, respectively (Table 2) [39]. CE also detected proximal small intestinal lesions in two-thirds of the patients inaccessible to ileocolonoscopy, but these results are of questionable clinical importance. Biancone et al. prospectively compared CE with SICUS for one year in 22 post-ileocolonic resection patients [40]. This recurrence was assessed, with ileocolonoscopy being the gold standard. Five of these patients were excluded due to intestinal strictures. Of the 17 who had all three investigations, recurrence was detected in 16 out of 17 by both CE and ileocolonoscopy, while SICUS detected recurrence in all the patients (one false positive) (Table 2) [40].

**Table 2:** Summary of the included studies highlighting the significance of CE in postoperative Crohn’s disease IC: Ileocolonoscopy; CE: Capsule endoscopy; Sens/Spec: Sensitivity/Specificity; SICUS: Small intestine contrast ultrasonography.

References	Compared modality	n	Results	
Beltrán et al. [36]	IC	19	IC recurrence: 25%	CE recurrence: 68%
Bourreille et al. [39]	IC	32	IC Sens/Spec: 90%/100%	CE Sens/Spec: 62%-76%/90%-100%
Biancone et al. [40]	SICUS	22	SICUS recurrence: 100%	CE recurrence: 94%

While most of these studies seemed to show that CE is indeed a relatively useful diagnostic tool with one of these quoting a higher detection rate, the majority of studies showed no significant advantage in its use when compared to other tools. It should also be noted that a major limitation of these studies is the sample size, and thus statistical significance was not met.

Inference of capsule endoscopy in the management of Crohn's disease

In clinical practice, CE has often been used as a means of monitoring treatment, thus influencing the need for further management. CE is highly dependent on its ability to detect CD lesions, as discussed earlier, among other factors.

Kopylov et al., in a retrospective study of 187 patients, reported that 52% of these patients had a change in treatment as a result of their CE outcomes (Table 3) [41]. In most patients (82%), anti-inflammatory medication was started or intensified, biologic therapy was used 30% of the time, immune-modulatory therapy was used 36% of the time, and surgical intervention was used 2% of the time [41]. Dussault et al., in a similar study of 71 patients with established CD, evaluated the influence of CE on treatment [42]. This was a retrospective study, and these patients had undergone CE for a variety of reasons; 45% of these patients were found to have moderate endoscopic lesions, while 17% had severe endoscopic lesions recorded [42]. It was found that treatment was modified in 54% of patients three months post-CE, and 27 of these patients were commenced on new medications (Table 3) [42]. These changes in management were significantly more significant in those with severe endoscopic lesions (75%) than those with moderate lesions (53%) [42]. This study's findings can be compared along the lines of another retrospective study conducted for seven years by Long et al. [43]. This more extensive study involved 128 CE studies of symptomatic IBD, with four excluded. Eighty-six of these were found to have CD, with 62% of them requiring a change in medical therapy three months post-CE. Also, severe endoscopic findings were linked with a switch in therapy [43].

**Table 3:** Summary of the included studies for indicating the inference of CE in the management of Crohn's disease CE: Capsule endoscopy.

References	n	CE findings	Change in management
Kopylov et al. [41]	187	Moderate-to-severe: 45%	52%
Dussault et al. [42]	71	Moderate: 45%	54%
		Severe: 17%	75%
Long et al. [43]	128	Moderate: 30.3%	51%
		Severe: 47.6%	73%
Min et al. [44]	50	86%	75%

The role of CE in influencing treatment decisions in children has also been reported. Min et al. studied whether CE would affect pediatric treatment [44]. CE was indicated in 50 patients with active CD and poor growth, 16 patients with indeterminate colitis (IC), and 17 with suspected IBD. One year before and after CE, the treatment and clinical outcomes were documented. The CE results were abnormal in the vast majority of patients with CD (86%) (Table 3) [44]. Escalation of treatment was necessary for 75% of patients (Table 3). These individuals' growth characteristics, clinical scores, and laboratory markers improved statistically significantly one year after CE. Since most people requiring an increase in dosage experienced poor growth or active symptoms, CE seemed to act as a supplement and not a substitute.

From these studies, it can be deduced that CE has been found to play a significant role in modifying therapy. It should be highlighted, however, that these studies are retrospective and thus not ideal. Also, there is a paucity of data, and it is clear that there have not been enough studies assessing the influence CE has on the treatment plans of CD patients.

Complications

CE is a relatively safe procedure; rare reports of complications support this. Its perception as being non-invasive has contributed to its appeal among patients and clinicians. However, certain complications arise as a result of the procedure, and they have been divided into clinical and technical complications [45]. Of these complications, by far, the most important and common is capsule retention. This has been defined as a capsule that remains in the gut lumen for a minimum of two weeks unless surgically or endoscopically removed or if it passes as a consequence of medical therapy [46]. This is a major worry not just for patients but also for professionals. Thankfully, capsule retention is relatively uncommon, with studies reporting a range of 1%-2% [47-49]. A comprehensive analysis encompassing 227 publications with 22,840 capsule trials reported a total retention rate of 1.4% [50]. However, for established CD, the overall retention rate was 2.6% [50]. This higher rate of retention can be attributed to the increased likelihood of intestinal strictures in CD.

Capsule retention can affect any area of the digestive system and remains undetected most of the time. However, symptomatic bowel obstruction might ensue, necessitating surgical or endoscopic excision of the afflicted capsule. In rare situations, intestinal perforation has been reported [51-54]. Despite this, some argue that capsule retention could be positive as it helps pinpoint the site of stenosis, thus aiding diagnosis and management [17,55].

Currently, the best clinical practice is to apply a permeability test once intestinal stricture is suspected. An example of such a test is applying a patency capsule (Agile Patency System, Given Imaging Ltd., Yoqneam, Israel). These are dissolvable capsules that are usually of the same size as the capsule used in CE, and the FDA approved them in 2006 [38,56,57]. They are uniquely designed to dissolve after 40 hours of interaction with digestive fluids. A radio-opaque substance, timer, and radiofrequency identification tag are also embedded, allowing it to be easily identified using a portable radiofrequency detector. These features make it possible to pinpoint the location of the stenosis and thus avoid capsule retention.

Other reported complications that could arise include but are not limited to technical complications such as short-life capsule batteries, downloading failure, failure of the localization software, recording gaps, and inability to activate the capsule [49]. These are issues that arise as a result of faults or inadequacies with the basic CE system, which could vary from the antenna on the capsule to the computer used to view recordings. There is, however, not enough evidence of their influence on the diagnosis.

Future prospects

With the introduction of artificial intelligence (AI) in clinical investigations, bowel endoscopy has evolved dramatically, notably in identifying and describing neoplastic and preneoplastic lesions. Several recent research has shown that AI-assisted endoscopy may diagnose CD.

While CE has been shown to be effective in its role as a counterpart to traditional methods of imaging in the diagnosis and treatment of CD, the time-consuming nature of interpreting films has been noted as a critical disadvantage of using CE with studies quoting a mean of 45-60 minutes [58-60]. User reliance further emphasizes this as the tired human eye misses tiny results that might be crucial in reaching a diagnosis. AI is a rapidly developing application that could help solve this problem alongside various others. Asides from this, AI-assisted endoscopy can be indirectly used for the localization of capsules by estimation of transit time and length of bowel. While some studies have stated this to be largely inaccurate, there is some hope that with improved technology, it would be possible [61-64].

Ding et al. retrospectively compared AI-assisted viewing of CE images (5000 patients) to manual reading [65]. AI-assisted reading was shown to be more sensitive and specific to human review with 99.9% specificity and sensitivity. Fan et al. similarly assessed this by integrating thousands of CE images [66]. They reported similar findings with ulcer and erosion detection sensitivity of 96.80% and 93.67%, specificity of 94.79% and 95.98%, and a commendable accuracy of 95.16% and 95.34%, respectively [66]. Aoki et al., in a retrospective study, examined 20 CE videos and compared the findings with AI-assisted technology [67]. It was reported that the AI-assisted reading time was much shorter (3.1 minutes) compared to the manual reading time (12.2 minutes) [67]. Furthermore, it was shown that the detection rate of mucosal breaks was not significantly reduced (87% and 84% detected by AI-assisted and manual reading, respectively) [67].

This definitely sounds exciting with many prospects; however, there is still some work to be done in this field. Most studies reviewed were retrospective studies; moving forward, prospective, multicentered studies would be needed to further provide credibility to the use of AI in CE.

Limitations

The role of CE in the management of CD is pervasive, with applications in nearly every step. However, our literature review does not examine specific aspects of this, such as the impact of standardized diagnostic criteria in CE. It also neglects to address the importance of CE in detecting mucosal repair.

## Conclusions

The essence of this review was to evaluate the pertinence of CE not only as a diagnostic tool in the management of CD but also the effect it has on further treatment plans. We also examined the current and possible forthcoming developments in its application. These studies agree that CE is more appropriate and specific in the diagnosis of small bowel lesions when compared to traditional imaging modalities, thus nullifying the need for multiple tests. Furthermore, we saw that findings post-CE had been shown to influence the treatment options in both the adult and pediatric population with CD. The clinical significance of this article is to provide a much-needed review of the relevance of CE in the management of CD. This would help leverage the many advantages, such as being more patient-friendly and cost-effective compared to other available modalities. Increased acceptance of CE would go a long way in decreasing morbidity and improving patients' quality of life. This review would also add to the increasing evidence of medical literature that presents CE's importance. While it may have some peculiar complications, studies have shown that innovations such as AI-assisted endoscopy could significantly improve its efficiency and eliminate some of its disadvantages. Overall, while some literature has assessed the role of CE in the management of CD, they are mostly statistically insignificant and unlikely to encourage a consensual acceptance of CE in clinical practice. More in-depth prospective studies with larger sample sizes should be performed to establish the role of CE in CD.
